# Optimizing the CRISPR/Cas9 system for genome editing in grape by using grape promoters

**DOI:** 10.1038/s41438-021-00489-z

**Published:** 2021-03-01

**Authors:** Chong Ren, Yanfei Liu, Yuchen Guo, Wei Duan, Peige Fan, Shaohua Li, Zhenchang Liang

**Affiliations:** 1 Beijing Key Laboratory of Grape Science and Enology, and CAS Key Laboratory of Plant Resources, Institute of Botany, The Innovative Academy of Seed Design, The Chinese Academy of Sciences, 100093 Beijing, People’s Republic of China; 2 University of Chinese Academy of Sciences, 100049 Beijing, People’s Republic of China

**Keywords:** Biotechnology

## Abstract

The efficacy of the CRISPR/Cas9 system in grapevine (*Vitis vinifera* L.) has been documented, but the optimization of this system, as well as CRISPR/Cas9-mediated multiplex genome editing, has not been explored in this species. Herein, we identified four VvU3 and VvU6 promoters and two ubiquitin (UBQ) promoters in grapevine and demonstrated that the use of the identified VvU3/U6 and UBQ2 promoters could significantly increase the editing efficiency in grape by improving the expression of sgRNA and *Cas9*, respectively. Furthermore, we conducted multiplex genome editing using the optimized CRISPR/Cas9 vector that contained the conventional multiple sgRNA expression cassettes or the polycistronic tRNA-sgRNA cassette (PTG) by targeting the sugar-related tonoplastic monosaccharide transporter (TMT) family members *TMT1* and *TMT2*, and the overall editing efficiencies were higher than 10%. The simultaneous editing of *TMT1* and *TMT2* resulted in reduced sugar levels, which indicated the role of these two genes in sugar accumulation in grapes. Moreover, the activities of the VvU3, VvU6, and UBQ2 promoters in tobacco genome editing were demonstrated by editing the phytoene desaturase (*PDS*) gene in *Nicotiana benthamiana* leaves. Our study provides materials for the optimization of the CRISPR/Cas9 system. To our knowledge, our simultaneous editing of the grape *TMT* family genes *TMT1* and *TMT2* constitutes the first example of multiplex genome editing in grape. The multiplex editing systems described in this manuscript expand the toolbox of grape genome editing, which would facilitate basic research and molecular breeding in grapevine.

## Introduction

The advent of the clustered regularly interspaced short palindromic repeats (CRISPR)/CRISPR-associated protein 9 (Cas9) system has been revolutionizing genome editing and genetic therapy^1,2^. In recent years, CRISPR/Cas9 has been more rapidly and widely applied in numerous plant species due to its simplicity, high efficiency, and versatility compared with previous genome editing technologies^3–5^. The most popular CRISPR/Cas9 system was initially derived from the adaptive immune system in *Streptococcus pyogenes*^6^, and an engineered system in which a single guide RNA (sgRNA) and the Cas9 nuclease are needed for genome editing^1^. The Cas9 nuclease can produce double-stranded DNA breaks, whereas the sgRNA specifically directs the Cas9 protein to its complementary DNA target site through RNA–DNA base pairing^1^. The expression of sgRNA and *Cas9* has been shown to influence the editing efficiency of the CRISPR/Cas9 system^7–10^.

In the CRISPR/Cas9 system, the expression of *Cas9* is generally driven by an RNA polymerase II (Pol II) promoter. During the early applications of the CRISPR/Cas9 system in plants, the CaMV35S promoter was often used to express the *Cas9* gene^10–12^. Recently, plant ubiquitin (UBQ) promoters have been isolated and employed in the CRISPR/Cas9 system in place of the 35S promoter^13–15^. The Pol III promoters of small nuclear RNA (snRNA) genes, such as U3 and U6, are commonly used to drive sgRNA expression in plants and animals^16,17^. U6 snRNAs participate in the intron splicing of pre-mRNA in the nucleus^18^, whereas U3 snRNAs are involved in pre-rRNA processing^19,20^. Unlike other Pol III-transcribed genes that contain intragenic promoter elements, U6 snRNA genes only possess a conserved TATA box and the upstream sequence element (USE) in their promoters^18,21^. In contrast, the promoter structures of U3 snRNA genes vary among different species, particularly metazoans^20^. In higher plants, however, a canonical TATA-like box and well-conserved proximal USE are present in the U3 promoters^20,21^. Both of these promoters are capable of producing high levels of sgRNA, which typically have a length of ~200 nucleotides^22^. In general, the Arabidopsis U6 (AtU6) and AtU3 promoters are extensively used in CRISPR/Cas9 vectors for genome editing in dicot plants, whereas the rice (*Oryza sativa*) U6 (OsU6) and OsU3 promoters are primarily applied in genome engineering in monocot plants^16,23^. Many CRISPR/Cas9 toolboxes have been developed and optimized based on the Pol III promoters isolated from the two model plant species^16,21,23^. Recently, plant species-specific U6 promoters for CRISPR/Cas9-mediated genome editing were adopted in soybean^8^, cotton^10,24^, apple^25^, and wheat^26^, and these studies revealed that the use of plant species-specific Pol III promoters could contribute to increased sgRNA levels and thus, result in enhanced editing efficiencies^8–10^. However, the identification and application of plant species-specific Pol III promoters have not been fully explored, and U3 and U6 promoters in other plant species are expected to be characterized during the development and optimization of CRISPR/Cas9 systems for these species. As a fruit crop that is widely cultivated worldwide, grape (*Vitis* ssp.) is economically important. CRISPR/Cas9-mediated genome editing in grape (*Vitis vinifera* L.) was first reported in 2016 (ref. ^27^), and since then, the AtU6 or AtU3 promoters have been used to drive the expression of sgRNAs during grape genome editing^27–31^. Nonetheless, the grape Pol III promoter has not yet been identified.

Multiplex genome editing can be achieved by stacking multiple sgRNAs in a single CRISPR vector^13^. The simplest and most common approach involves the use of multiple U3 and/or U6 promoters for the expression of different sgRNAs^32^. Additionally, approaches based on self-cleaving ribozymes (RZ), tRNA, or Csy4 have also been used to produce multiple sgRNAs^33,34^. Multiplex genome editing, which enables the simultaneous targeting of several related or unrelated genes, has been applied in many plant species^35^. In this method, different alleles of the same gene, homoeoalleles in polyploid plants, or even a gene family could be simultaneously edited through multiplex gene editing^35,36^. The greatest potential of multiplex genome editing has been shown by editing different genes involved in the control of distinct traits in plants. For instance, the CRISPR/Cas9-mediated targeting of multiple genes that control the time of flowering and plant morphology can accelerate the domestication of wild tomato or directly develop customized tomato for urban agriculture^37,38^. In recent years, an increasing number of reported studies have implemented targeted genome editing in grape, and almost all the studies were performed with one or several sgRNAs targeting single genes^27–31,39^. The simultaneous editing of different genes of interest could improve different traits of grapes, which would be extremely significant for shortening the period of grape breeding. However, CRISPR/Cas9-mediated multiplex genome editing involving different genes has not been explored in grape.

In the present study, we first identified four VvU3 and VvU6 promoters and two UBQ promoters in grape and investigated their efficacy and efficiency in genome editing by targeting the grape phytoene desaturase (*PDS*) gene. The use of grape VvU3/U6 and UBQ2 promoters can clearly improve the editing efficiencies in grape cells and stable transgenic plants by increasing the expression of sgRNA and *Cas9*, respectively. We then optimized the CRISPR/Cas9 system using the VvU6 and UBQ2 promoters instead of the AtU6 and 35S promoters, respectively, and developed a multiplex genome editing system containing the traditional multiple sgRNA (multi-sgRNA) expression cassettes or the polycistronic tRNA-sgRNA cassette (PTG). The simultaneous editing of the grape tonoplastic monosaccharide transporter (TMT) genes *TMT1* and *TMT2* demonstrated the efficacy of the two systems. Additionally, the successful editing of the tobacco *NbPDS* gene suggests that the identified VvU3 and VvU6 promoters, as well as the UBQ2 promoter, might be applied in other dicot plants.

## Results

### **I**dentification of U3, U6, and UBQ promoters in grapevine

**Fig. 1:**
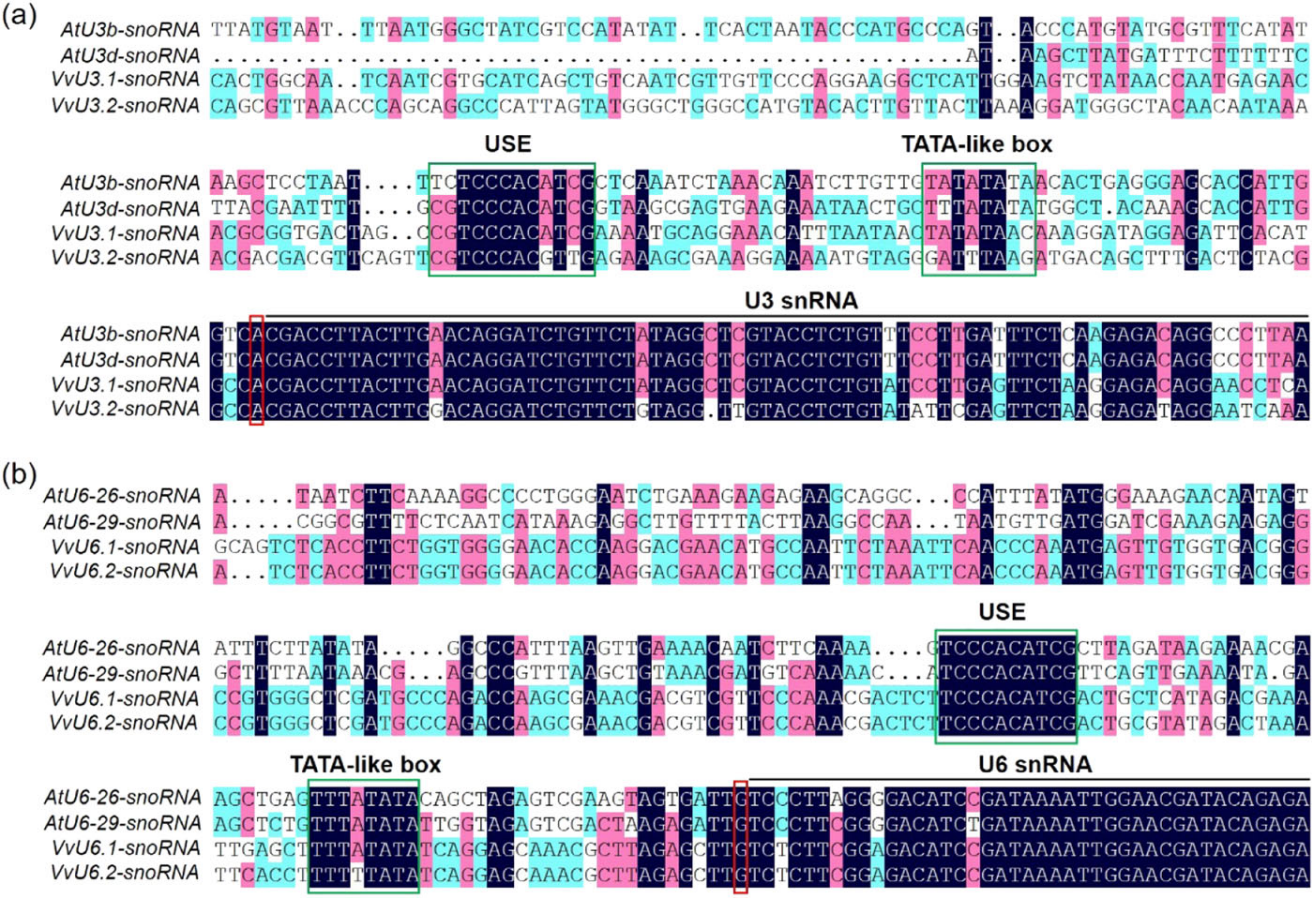
Analysis of grape VvU3 and VvU6 promoter sequences. Multiple alignments of the grape and Arabidopsis U3 (**a**) and U6 (**b**) snRNA genes are shown. Black lines indicate snRNA transcripts. The conserved elements USE (upstream sequence element) and TATA-like box are indicated by green boxes. The nucleotides “A” (adenine) and “G” (guanine) recognized by the U3 and U6 promoters for transcription initiation are labeled in red boxes. Different colors denote different levels of sequence identity. The nucleotides with 100% identity are highlighted in black, and those with ≥75% and ≥50% identity are highlighted in red and blue, respectively. The multiple sequence alignments were performed using DNAMAN software

In previous studies on genome editing in grape, AtU6 and 35S promoters were commonly used to drive the expression of sgRNAs and *Cas9*, respectively^27,29,31,40^. In an effort to optimize the CRISPR/Cas9 system in grape, we conducted BLAST searches of the grape genome (*V. vinifera* L.) using the Arabidopsis *AtU6-26* and *AtU3b* genes as the queries, and identified four *VvU6* and *VvU3* snRNA genes showing high sequence similarities to the queries (Table 1). The two *VvU6* (*VvU6.1* and *VvU6.2*) and two *VvU3* (*VvU3.1* and *VvU3.2*) snRNA genes exhibited relatively conserved snRNA transcript sequences compared with the corresponding *AtU6* and *AtU3* genes. However, the promoter regions of these genes were divergent between the two species, with the exception of the USE and TATA-like box, which are required for transcription (Fig. 1a). The presence of the USE and TATA-like box in the promoters of the *VvU3* and *VvU6* genes suggests that these Pol III promoters might be involved in effective transcription. We therefore cloned the promoter regions upstream of the transcription initiation site of the four *VvU3* and *VvU6* genes as the VvU3 and VvU6 promoters for subsequent experiments (Table 1 and Supplementary Fig. S1). In addition, we screened two grape *UBQ* genes that are constitutively expressed in grapevine (Table 1 and Supplementary Fig. S2). In this study, 748-bp and 845-bp DNA fragments upstream of the start codon “ATG” were amplified as putative UBQ promoters (Supplementary Fig. S3).

**Table 1:** Amplified Pol II and Pol III promoters in grape.

Promoter	Gene ID	Size (bp)
VvU3.1	ENSRNA049469468	393
VvU3.2	ENSRNA049467827	483
VvU6.1	ENSRNA049469148	425
VvU6.2	ENSRNA049469157	591
UBQ1	VIT_19s0177 g00040	748
UBQ2	VIT_19s0177 g00070	845

### **A**ssay of the activities of the VvU3, VvU6, and UBQ promoters via transient expression in tobacco and grapevine leaves

**Fig. 2:**
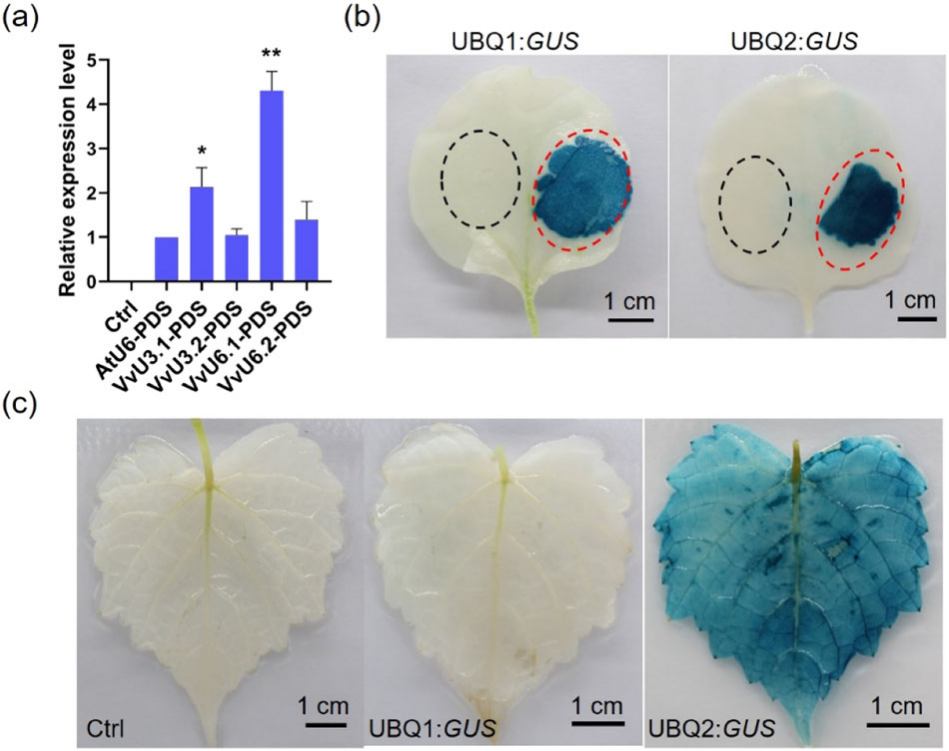
Assay of the activities of the identified VvU3, VvU6, and UBQ promoters. **a** Relative expression of grape *PDS* sgRNA driven by the VvU3 and VvU6 promoters in *Nicotiana benthamiana* leaves. The CRISPR vectors were infiltrated into *N. benthamiana* leaves for transient expression, and 3 days after infiltration, the sgRNA expression level relative to that of the *Cas9* gene was determined by quantitative real-time PCR (qRT-PCR). Non-infiltrated plants were used as a negative control (ctrl). The data are presented as the mean values ± SDs from three biological replicates; * and ** indicate significant (*P* < 0.05) and highly significant (*P* < 0.01) differences, respectively; **b** and **c** GUS staining of tobacco and *V. amurensis* leaves, respectively. The UBQ1:*GUS* and UBQ2:*GUS* constructs were infiltrated into *N. benthamiana* leaves, and the infiltrated regions are shown in red dotted ellipses. *Agrobacterium* cells containing no expression vector served as the control (ctrl), and the control regions are marked with black dotted ellipses. GUS staining was conducted 3 days after infiltration

To investigate whether the amplified VvU3 and VvU6 promoters could drive the expression of sgRNA, we used these Pol III promoters instead of the AtU6 promoter in the pCACRISPR/Cas9 vector^27^ containing a sgRNA targeting the grape *PDS* gene. The CRISPR vectors were then introduced into *Nicotiana benthamiana* leaves via *Agrobacterium*-mediated infiltration for transient expression. The expression of *PDS* sgRNA was inspected by quantitative real-time PCR (qRT-PCR). According to the results, the VvU3 and VvU6 promoters successfully promoted the expression of *PDS* sgRNA in tobacco leaves, and the sgRNA expression levels driven by the VvU3 and VvU6 promoters were comparable or higher than those coordinated by the AtU6 promoter (Fig. 2a).

Similarly, the activities of the UBQ promoters were first assessed in tobacco leaves, and the β-glucuronidase (*GUS*) gene fused to the UBQ promoters was used as the reporter. As shown in Fig. 2b, both UBQ1 and UBQ2 promoted the expression of the *GUS* gene in tobacco leaves, and the activity of UBQ2 was higher than that of UBQ1, as indicated by the finding that the leaf region infiltrated with the UBQ2:*GUS* construct exhibited stronger GUS staining intensity compared with the region transformed with the UBQ1:*GUS* vector (Fig. 2b). However, no GUS staining was observed in the grapevine leaves after infiltration with the UBQ1:*GUS* construct, whereas strong GUS staining was observed with the UBQ2 promoter (Fig. 2c and Supplementary Fig. S4). Based on these results, the UBQ2 promoter was selected for further study.

### **T**he use of the VvU3/U6 and UBQ2 promoters contributes to genome editing in grape

**Fig. 3:**
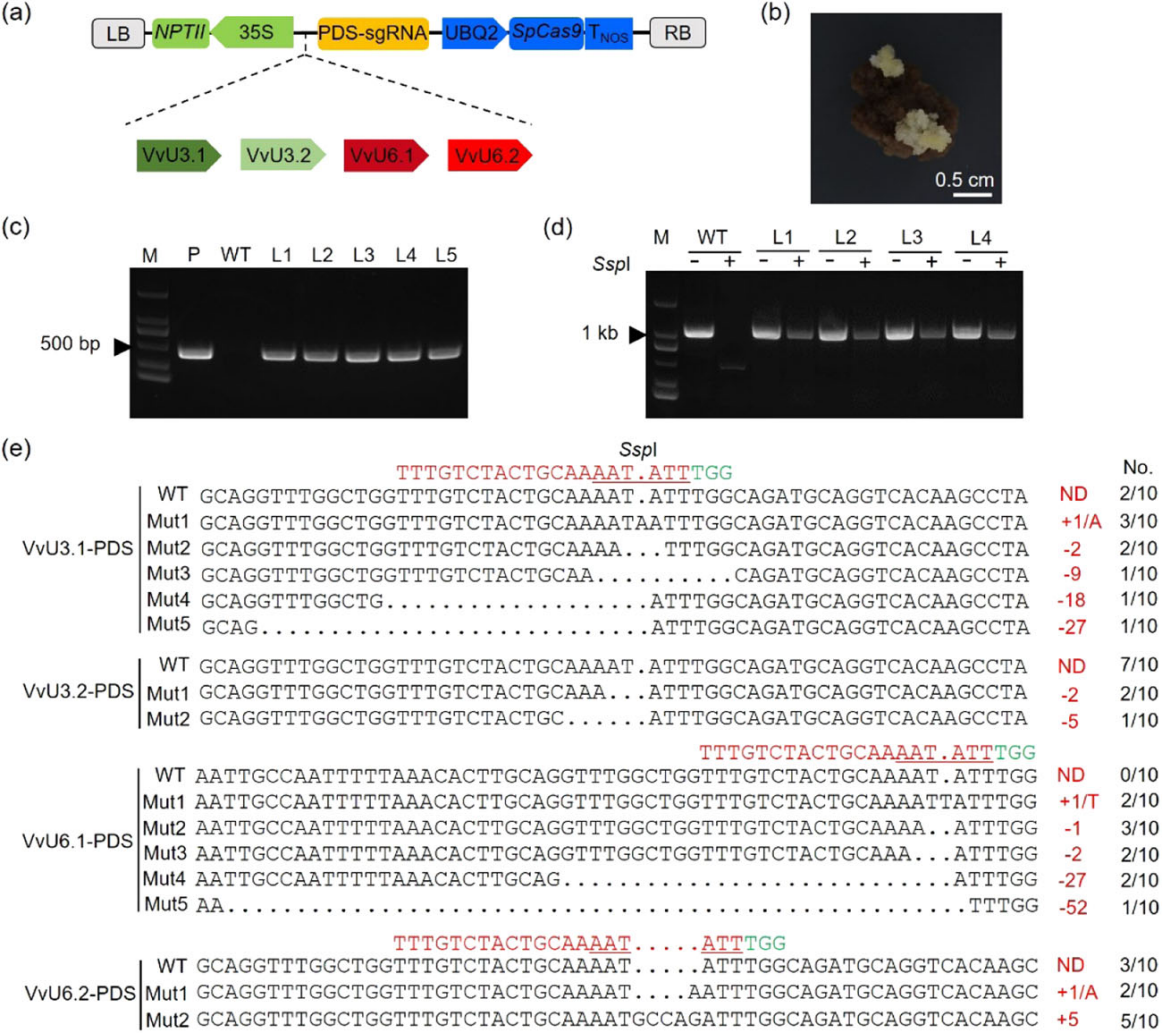
Targeted mutations generated using the VvU3, VvU6, and UBQ2 promoters. **a** Schematic illustration of sgRNA/Cas9 vectors. The cloned VvU3 and VvU6 promoters were used instead of the AtU6 promoter, whereas the CaMV35S promoter that drives the expression of *Streptococcus pyogenes Cas9* (*SpCas9*) was replaced by the UBQ2 promoter. *NPT*II, neomycin phosphotransferase gene; T_NOS_, terminator of nopaline synthase gene; LB, left border; RB, right border. **b** Resistant 41B cells generated on selective medium. **c** PCR identification of exogenous T-DNA insertions. *Cas9*-specific primers were used to identify the T-DNA in 41B cells. The CRISPR vector and wild-type (WT) cells were used as positive (P) and negative controls, respectively. Lanes 1–5 (L1–5) represent samples from VvU3.1-PDS-, VvU3.2-PDS-, VvU6.1-PDS-, VvU6.2-PDS-, and AtU6-PDS-containing cells, respectively. M, DNA marker. **d** Restriction (RE)/PCR assay. The target sequence of the grape *PDS* gene contained an *Ssp*I enzyme site, genomic DNA (gDNA) prepared from VvU3.1-PDS- (L1), VvU3.2-PDS- (L2), VvU6.1-PDS- (L3), and VvU6.2-PDS-containing (L4) cells was treated with *Ssp*I, and mutated sequences were enriched by PCR due to the disruption of the enzyme site after editing. The WT gDNA was used as a negative control. +, *Ssp*I digestion; −, no digestion. **e** Sequencing results of the target sequences. The enriched target sequences after RE/PCR shown in **d** were cloned into the pLB vector for Sanger sequencing. For each sample, a total of 10 clones were sequenced. The mutation type and corresponding number of clones are shown in red and black on the right, respectively. ND, not detected. WT, wild-type sequences. Mut, mutated sequences. The PAM sequence is indicated in green, and the *Ssp*I enzyme site is underlined

It has been reported that using plant species-specific promoters could increase the expression of sgRNA/Cas9, resulting in enhanced editing efficiencies^8–10^. To determine whether the use of grape VvU3/U6 and UBQ2 promoters results in improved genome editing efficiency in grape, we constructed optimized CRISPR/Cas9 vectors based on the backbone of the pCAMBIA2300 vector using the grape UBQ2 promoter to drive the expression of *Cas9* and the VvU3/U6 promoters to express *PDS* sgRNA (Fig. 3a). The optimized CRISPR vectors were designated pCA-UBQ2-Cas9-VvU3/U6-PDS and introduced into embryogenic cells derived from 41B grapevine rootstock via *Agrobacterium*-mediated transformation, and kanamycin-resistant cells were obtained after antibiotic-dependent selection (Fig. 3b). Exogenous T-DNA insertions were identified by PCR using *Cas9*-specific primers (Supplementary Table S1). The results revealed that the kanamycin-resistant cells contained the exogenous *Cas9* gene (Fig. 3c), which suggested that the CRISPR vectors had been successfully introduced into the grape cells. We therefore checked the target site of the *PDS* gene in these transgenic cells. Given that the presence of unedited (or wild-type) sequences from untransformed cells could influence the identification of mutated sequences, we employed the restriction enzyme (RE)/PCR approach for the detection of mutagenesis in the *PDS* gene. The isolated genomic DNA (gDNA) from the grape cells was treated with the *Ssp*I restriction enzyme to digest the wild-type sequences, and the mutated DNA sequences, which are recalcitrant to enzyme digestion, could be enriched via subsequent PCR amplification (Fig. 3d). The desired bands were purified and sent for Sanger sequencing. As expected, indel (deletions or insertions) mutations at the target site were observed in the VvU3-PDS and VvU6-PDS transgenic cells (Fig. 3e). Most of the mutations were short nucleotide (<10 bp) insertions or deletions (Fig. 3e), which is consistent with our previous results obtained using the AtU6 promoter^40^. In addition, mutations consisting of ≥18-bp deletions were also observed in the VvU3.1-PDS and VvU6.1-PDS transgenic cells (Fig. 3e). These results demonstrated the efficacy of the VvU3, VvU6, and UBQ2 promoters in CRISPR/Cas9-mediated genome editing in grape.

**Fig. 4:**
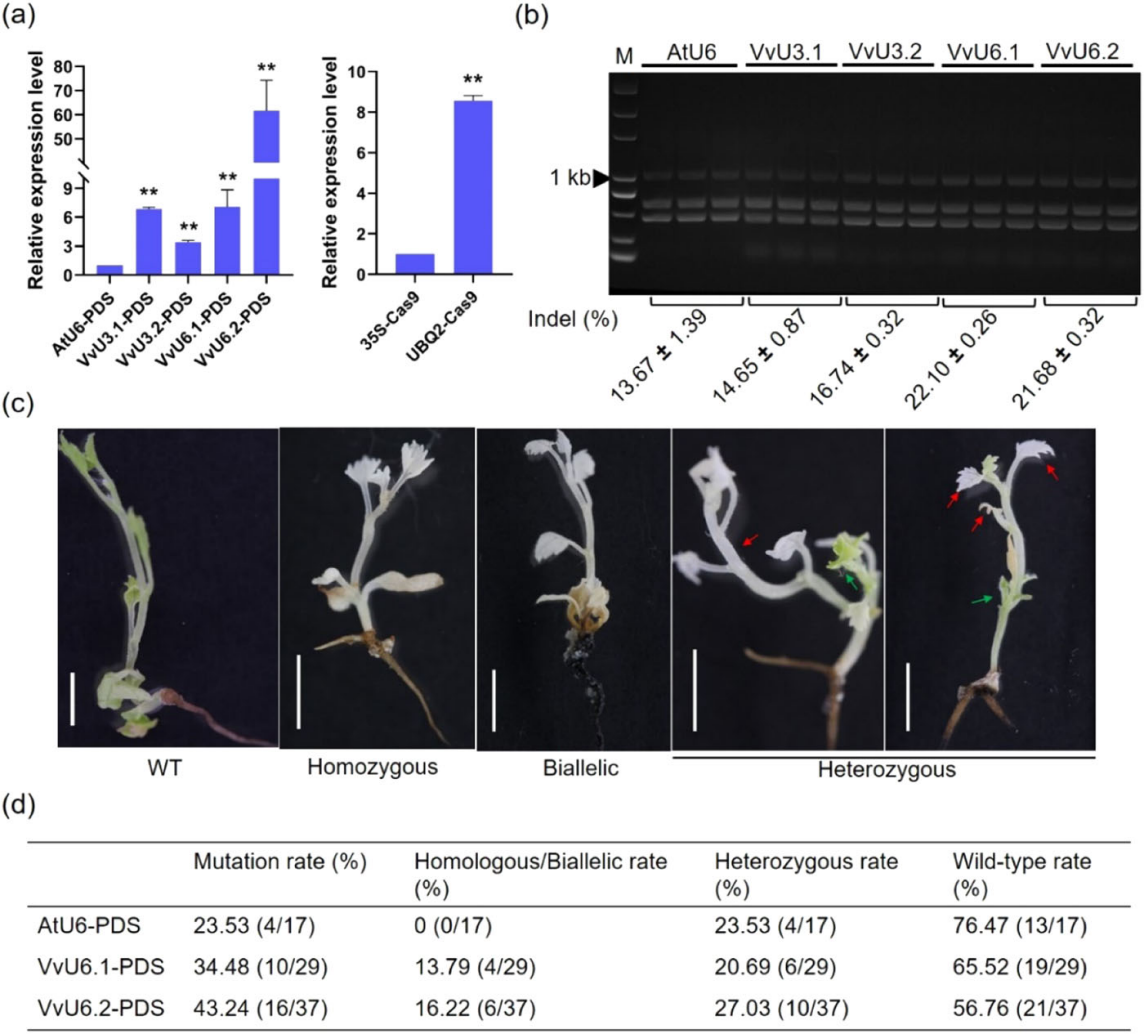
High-efficiency genome editing in transgenic grapevine plants. The most commonly used AtU6 and 35S promoters served as controls in the analysis of the efficiency of grape promoters. **a** Expression profiles of *PDS* sgRNA and *Cas9* in 41B cells. The relative expression levels of sgRNA and *Cas9* were determined by qRT-PCR, and the AtU6-PDS and 35S-Cas9 constructs were used as controls. The data are shown as the mean values ± SDs from three biological replicates; ** indicates a highly significant (*P* < 0.01) difference. **b** Restriction fragment length polymorphism (RFLP) analysis of the editing efficiency. The PCR amplicons were treated with the *Ssp*I enzyme and then separated on an EtBr-stained agarose gel (1.5%). The digested bands are indicated by red triangles. The intensities of the bands were quantified with ImageJ. The intensity of an undigested band relative to the whole band was calculated as an indel mutation^40^. The data are shown as the mean values ± SDs from three technical replicates. **c** Albino phenotypes of grapevine plants after targeted editing. Several albino plants are shown as examples. The albino parts of the heterozygous plants are indicated by red arrows and the green parts are denoted by green arrows. Scale bars: 0.5 cm. **d** Overview of site-specific editing using the U6 promoters in grapevine

The expression of sgRNA and *Cas9* in transgenic grape cells was measured by qRT-PCR, and the results showed that the transcript levels of the *PDS* sgRNA driven by the VvU3 and VvU6 promoters were higher (>3-fold) than those obtained using the AtU6 promoter (Fig. 4a). In addition to sgRNA expression, the transcript abundance of *Cas9* was significantly increased when using the UBQ2 promoter (Fig. 4a). The editing efficiencies in the transgenic cells were assessed using the restriction fragment length polymorphism (RFLP) method. The target fragment of the *PDS* gene was amplified from the AtU6-, VvU3.1-, VvU3.2-, VvU6.1-, and VvU6.2-PDS transgenic cells by PCR, and the PCR products were digested with the *Ssp*I enzyme. As mentioned above, successful targeted editing destroyed the *Ssp*I restriction enzyme site, resulting in accumulation of the mutated sequences. The indel frequency can be calculated according to the intensity of undigested PCR bands^40,41^. The RFLP analysis showed that the use of the VvU3 and VvU6 promoters resulted in higher editing efficiencies, and the indel frequencies ranged from 14.65% to 22.10% (Fig. 4b and Supplementary Table S2). In contrast, the editing efficiency obtained with the AtU6 promoter was 13.67% (Fig. 4b and Supplementary Table S2).

Interestingly, the editing efficiencies with the VvU3 promoters were slightly lower than those obtained with the VvU6 promoters (Fig. 4b). One possible reason is that the eukaryotic U3 promoter requires adenine (A) for transcription initiation, whereas the U6 promoter recognizes guanine (G) to initiate transcription. The additional “A” or “G” at the 5ʹ ends of the mature sgRNA could affect the editing efficiencies^42,43^. Therefore, we adopted VvU6-PDS transgenic cells for plant regeneration, and albino plants were obtained as expected (Fig. 4c). According to the results, the mutation rates of VvU6.1-PDS (34.48%, 10/29) and VvU6.2-PDS (43.24%, 16/37) were higher than that of AtU6-PDS (23.53%, 4/17), and homologous or biallelic plants were obtained with the VvU6.1 and VvU6.2 promoters at rates of 13.79% and 16.22%, respectively (Fig. 4d and Supplementary Fig. S5). Collectively, these results suggested that the use of species-specific VvU3/U6 and UBQ2 promoters resulted in high editing efficiency in grapes.

### **H**igh-efficiency multiplex genome editing in grape using grape promoters

**Fig. 5:**
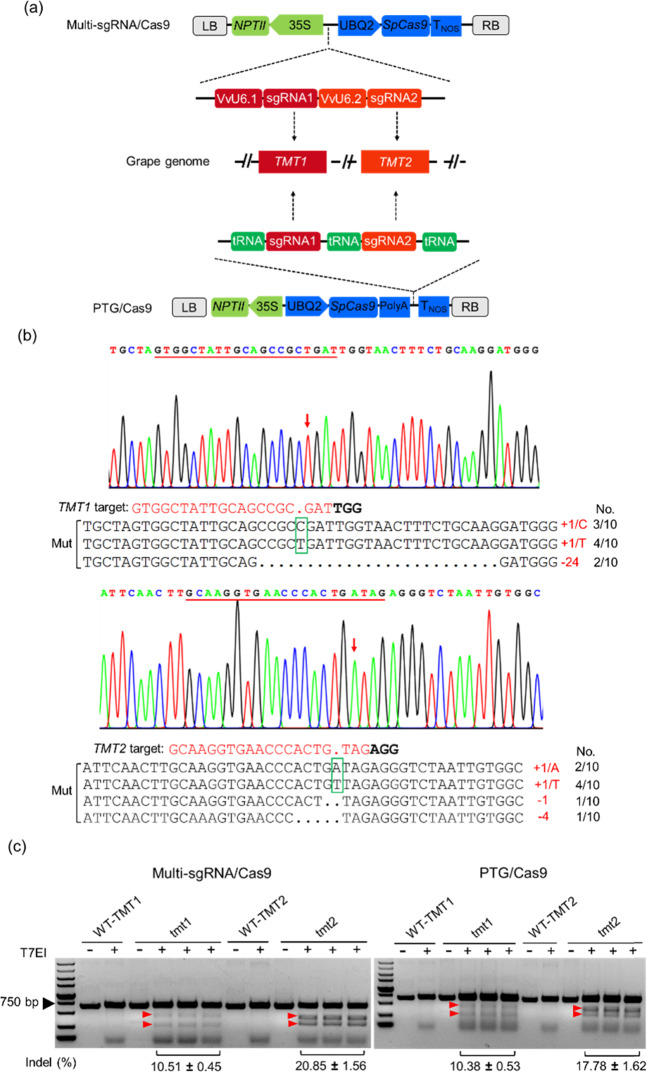
Multiplex editing in grape using multi-sgRNA/Cas9 and PTG/Cas9 systems. **a** Schematic diagrams of the multi-sgRNA/Cas9 and PTG/Cas9 vectors and the editing targets. **b** Sanger sequencing results of the editing at *TMT1* and *TMT2* target sites. The gDNA was extracted from transformed 41B cells for amplification of the target sequences. A total of 10 amplicons were sent for sequencing. A representative chromatogram and the mutated sequences identified for each target are shown. The target regions are underlined, and the mutation sites are indicated with red arrows in the chromatograms. The mutation types, as well as the corresponding number of sequences, are shown on the right. The inserted nucleotides are shown by green boxes. Mut, mutated sequences. **c** T7EI assay of the editing efficiencies at *TMT1* and *TMT2* target sites. The PCR amplicons containing the *TMT1* or *TMT2* target sequences were treated with the T7EI enzyme, and the digested products were separated on an EtBr-stained agarose gel (1.5%). The wild-type sequences of *TMT1* (WT-TMT1) or *TMT2* (WT-TMT2) served as controls. The intensities of the bands were quantified using ImageJ. The indel efficiencies calculated from the band intensities^40^ are shown as the mean values ± SDs from three technical replicates; +, T7EI digestion; −, no digestion

Given that the use of VvU3/VvU6 and UBQ2 promoters resulted in efficient genome editing, we hypothesized that these promoters can be used to express different sgRNAs for multiplex genome editing in grape. Multiplex genome editing enables researchers to study the functions of several related genes or even perform metabolic engineering in plants^36,44^. However, previous studies on genome editing in grape have primarily focused on single genes^27–31,39^, and editing studies involving different genes of interest have not been reported. To investigate the efficacy of the CRISPR/Cas9-based multiplex editing system in grape, we edited the *TMT* gene family in 41B grape cells, which have been successfully used to explore sugar uptake and accumulation^45^. Grape has three *TMT* genes, namely *TMT1* (VIT_18s0122g00850), *TMT2* (VIT_03s0038g03940), and *TMT3* (VIT_07s0031g02270), which show high similarity to their homologs in Arabidopsis^46^. However, the role of these three genes in sugar accumulation in grape has not been explored. By investigating the expression profiles of the three genes in different tissues or organs at different developmental stages, we found that *TMT1* and *TMT2* exhibited high transcript levels in grape berries, particularly at veraison and during ripening, whereas the expression of *TMT3* was poor or not detected in berries (Supplementary Figs. S6 and S7). Similar results were observed in 41B cells (Supplementary Fig. S8). These findings suggested that *TMT1* and *TMT2* play a vital role in sugar accumulation in vacuoles. Hence, we selected *TMT1* and *TMT2* as our target genes. Specific sgRNAs targeting *TMT1* and *TMT2* were designed and ligated to the VvU6.1 and VvU6.2 promoters, respectively. The two individual sgRNA expression cassettes were connected by overlapping PCR, and the combined multi-sgRNA expression cassette was used instead of the sgRNA expression cassette in the pCA-UBQ2-Cas9-VvU3/U6-sgRNA vector to generate the multiplex editing vector pCA-UBQ2-Cas9-VvU6-TMTs (Fig. 5a). Furthermore, we synthesized the polycistronic tRNA-TMT sgRNA cassette (Supplementary Fig. S9) and developed the PTG/Cas9 system, in which the expression of *Cas9*, sgRNAs, and tRNA was driven by the single UBQ2 promoter (Fig. 5a).

**Fig. 6:**
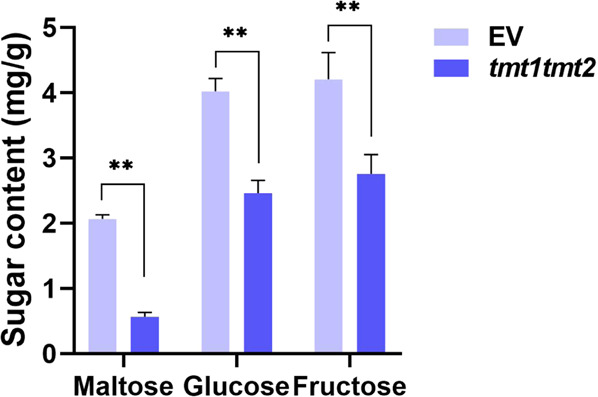
Sugar content in the *tmt1tmt2* cells. Edited cells obtained using the multi-sgRNA/Cas9 system were collected as examples for determination of the sugar content, and grape cells transformed with empty vector (EV) were used as a control. The data are shown as the mean values ± SDs from four biological replicates; ** indicates a highly significant (*P* < 0.01) difference

The constructed vectors were introduced into 41B cells. After transformation and antibiotic-dependent selection, transgenic grape cells were recovered for mutation identification. Both the multi-sgRNA/Cas9 and PTG/Cas9 systems could successfully induce the desired mutagenesis in the target genes. As shown in Fig. 5b, mutated sequences were detected in the amplicons of both the *TMT1* and *TMT2* target sequences. Single nucleotide insertions and nucleotide deletions were observed at the target sites (Fig. 5b), consistent with previous reports^27,31,40^. The T7EI assay revealed that the multi-sgRNA/Cas9 system resulted in editing efficiencies of 10.51% and 20.85% at the *TMT1* and *TMT2* target sites, respectively (Fig. 5c and Supplementary Table S2). Comparable mutation efficiencies were also generated using the PTG/Cas9 system at the *TMT1* (10.38%) and *TMT2* (17.78%) sites (Fig. 5c and Supplementary Table S2). Intriguingly, the *TMT2* sgRNA had a higher editing efficiency than the *TMT1* sgRNA in both the multi-sgRNA/Cas9 and PTG/Cas9 systems (Fig. 5c), which suggested that the sgRNA sequence had an effect on the editing efficiency. To evaluate the effect of *TMT1* and *TMT2* mutagenesis on sugar accumulation, we measured the sugar content in the edited 41B cells. Compared with the cells transformed with empty vector (EV), the contents of maltose, glucose, and fructose in the *tmt1tmt2* cells were significantly reduced (Fig. 6), which suggested that the mutation of *TMT1* and *TMT2* affected the extent of sugar accumulation in grape cells.

### **T**he VvU3/U6 and UBQ2 promoters are suitable for genome editing in tobacco

**Fig. 7:**
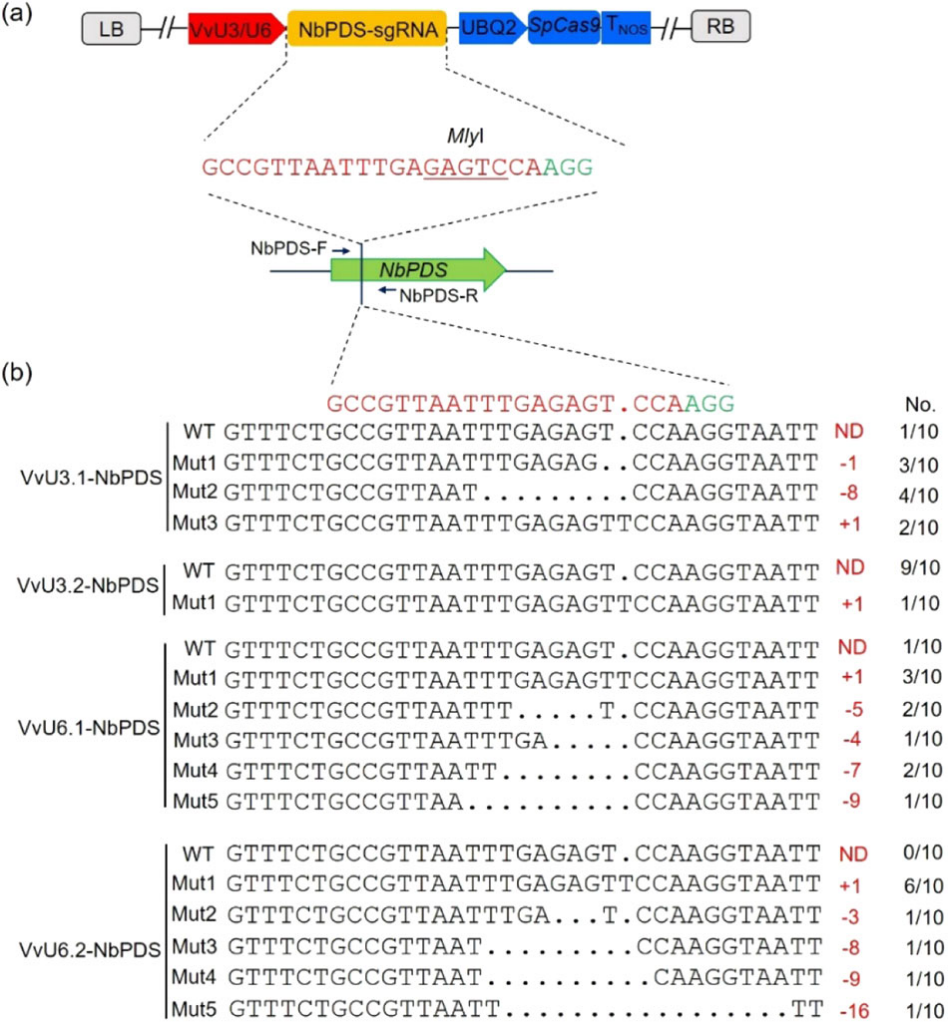
Targeted editing of the *NbPDS* gene in tobacco leaves using grape promoters. **a** Schematic diagram of the CRISPR vectors and target selection for tobacco genome editing. The primers NbPDS-F/R were used to amplify the target sequence of the *NbPDS* gene. The *Mly*I restriction enzyme site is underlined. **b** Sequencing results of targeted mutations in the *NbPDS*. The CRISPR vectors harboring the VvU3 and VvU6 promoters were infiltrated into *N. benthamiana* leaves for transient expression. The mutated sequences (Mut) were analyzed by RE/PCR and Sanger sequencing assays. The mutation types and corresponding number of mutated sequences are shown in red and black, respectively, on the right

As mentioned above, the VvU3/VvU6 and UBQ2 promoters can drive the expression of sgRNA and *GUS* reporter genes in *N. benthamiana* leaves (Fig. 2a, b), which suggests that these promoters might be used for genome editing in tobacco. To confirm the efficacy of the VvU3/U6 and UBQ2 promoters in tobacco genome editing, the grape *PDS* sgRNA in the pCA-UBQ2-Cas9-VvU3/U6-PDS vectors (Fig. 2a) was replaced by the *NbPDS* sgRNA (Fig. 7a). The modified vectors were infiltrated into *N. benthamiana* leaves for transient expression. The tobacco leaves were sampled for mutation identification. Sanger sequencing assays showed that the use of the VvU3/U6 and UBQ2 promoters resulted in successful targeted mutagenesis at the target site in the *NbPDS* gene (Fig. 7b), which confirmed the efficacy of the grape Pol II and Pol III promoters in tobacco genome editing.

## Discussion

As an RNA-guided DNA endonuclease system, sgRNA activity and the expression of sgRNA/Cas9 greatly influence the efficiency of CRISPR/Cas9-mediated genome editing^47,48^. The application of plant species-specific promoters is an effective strategy for improving the efficiency of genome engineering in plants^8,10^. To date, the widely used eukaryotic U3 and U6 promoters in monocot and dicot plants have been isolated from rice and Arabidopsis, respectively^21,23^. In grapevine, the AtU6 and 35S promoters are commonly used to drive the expression of sgRNA and *Cas9*, respectively, in genome editing^27,31,40^. In this study, we characterized four Pol III promoters (VvU3.1, VvU3.2, VvU6.1, and VvU6.2) and one Pol II promoter (UBQ2) in grape and verified their efficiency in grape genome editing by targeting the *PDS* gene (Fig. 3). We established that both the UBQ2 and VvU3/U6 promoters exhibited strong activities in promoting the expression of *Cas9* and sgRNA in grape (Fig. 4a), which resulted in high editing efficiencies (Fig. 4b, d). The results demonstrated that the use of species-specific promoters could increase the genome editing efficiency in grape.

In addition, the amplified VvU3 and VvU6 promoters can be used for the expression of several distinct sgRNA modules, which can direct the Cas9 protein to edit different targets simultaneously. This Pol III promoter-dependent strategy is commonly adopted for multiplex editing in plants^13,15,16,21^. In the present study, multiple sgRNA expression cassettes were assembled using the VvU6.1 and VvU6.2 promoters (Fig. 5a), because the first nucleotide of *TMT1* and *TMT2* sgRNAs is “G”. This multi-sgRNA/Cas9 system was demonstrated to be effective in grape, with editing efficiencies of 10.51% and 20.85% at the *TMT1* and *TMT2* targets, respectively (Fig. 5c). Individual sgRNA expression cassettes are relatively easy to construct, and several assembly methods have been developed^16,49^. However, the use of many promoters results in a large expression vector, and size limitation remains a challenge when expressing an increased number of sgRNAs^32^. Plant Pol III promoters generally require conserved elements of the USE and TATA-like box for effective transcription, and shortened U3 and U6 promoters containing only the USE, TATA box, and several artificial monocot-specific promoter elements have been developed for genome editing in rice^21^. Our identified VvU3 and VvU6 promoters contained the required USE and TATA-like box elements (Fig. 1), which suggested that the current promoters could also be optimized in the future. An alternative strategy for multiplex genome editing is expressing multiple sgRNAs from a single transcript^34^. In this case, endoribonuclease Csy4, tRNA, or RZs are generally employed to generate different sgRNAs^34^. However, *Csy4* should be coexpressed with an artificial sgRNA array when using the Csy4-based sgRNA expression method^34^. Furthermore, in rice, the RZ system exhibited relatively poor editing activity compared with the tRNA system^14^. The tRNA-based PTG system is a very efficient approach for the simultaneous editing of several genes of interest with high efficiencies in plants^36,50^. We also investigated the efficacy of the PTG/Cas9 system in grape, and the results showed that the PTG/Cas9 system was as effective as the multi-sgRNA/Cas9 system in editing the *TMT1* and *TMT2* genes (Fig. 5). The editing efficiencies using the PTG/Cas9 system were slightly lower than those obtained using the multi-sgRNA/Cas9 system (Fig. 5c), and the difference could be explained by different promoters or processing efficiencies of the primary transcript.

To date, the CRISPR/Cas9 system has been a powerful tool for crop improvement. For instance, the simultaneous editing of three different genes helps increase the yield and cold tolerance of rice^15^, and the engineering of three homoeoalleles in bread wheat confers resistance to powdery mildew^51^. In grapevine, however, the CRISPR/Cas9 system has not been fully exploited, and the application of this technology is restricted to single gene editing due to the limited CRISPR toolbox available. Multiple genes generally control the important traits involved in the quality of fruits of grape, such as sugar and organic acid accumulation^46,52^. TMTs are important sugar transporters that are reportedly associated with sugar accumulation in sugar beets and watermelon^53,54^. Of the three *TMT* genes in grape, *TMT1* and *TMT2* are thought to be involved in sugar accumulation in grape berries^46^. Knockout of the *TMT1* and *TMT2* genes in grape cells significantly reduced the sugar content (Fig. 6). The simultaneous editing of the grape *TMT1* and *TMT2* genes provides evidence that can be used to deduce the function of these two genes in sugar accumulation in grape and constitutes, to our knowledge, the first example of multiplex genome editing in grape.

In conclusion, our study provides effective and robust grape Pol II and Pol III promoters for the optimization of the CRISPR/Cas9 system, and these promoters increase the editing efficiency in grape. Moreover, the developed multiplex genome editing systems, including the multi-sgRNA/Cas9 and PTG/Cas9 systems, expand the toolbox of grape genome editing and can thus facilitate the application of CRISPR/Cas9 technology to the future study of functional genes and mutant phenotypes in grape.

## Materials and methods

### **P**lant materials and culture conditions

Grapevine plants of *V. vinifera* cv. Pinot Noir were grown in the germplasm resources garden at the Institute of Botany, the Chinese Academy of Sciences, Beijing, under natural conditions. The leaves were sampled for gDNA isolation and the prepared gDNA was used for promoter cloning. 41B (*Vitis vinifera* cv. Chasselas × *Vitis berlandieri*, a rootstock) cells were cultured in liquid glycerol-maltose (GM) medium as previously reported^40^. In vitro plants of *Vitis amurensis* were subcultured in half-strength Murashige & Skoog medium (PhytoTech). Seeds of *N. benthamiana* were sown in the soil. Both the *V. amurensis* and tobacco plants grew at 26 °C under long-day conditions (16-h light/8-h dark). Leaves of *V. amurensis* and tobacco plants were used for transient expression experiments.

### **C**loning of promoters and construction of CRISPR vectors

The Arabidopsis snRNAs *AtU3b* (AT5G53902) and *AtU6-26* (AT3G13855) and the *AtUBQ* gene (AT3G52590) sequences were used as queries to identify the grape *U3, U6*, and *UBQ* genes from the grape genome (http://plants.ensembl.org/Vitis_vinifera/Info/Index), respectively. Approximately 500–800-bp DNA fragments upstream of the transcription start sites of the identified genes were amplified and cloned into the pLB vector (TIANGEN) for sequencing. The correct plasmids of VvU3.1-pLB, VvU3.2-pLB, VvU6.1-pLB, VvU6.2-pLB, UBQ1-pLB, and UBQ2-pLB were kept as templates for subsequent PCR.

To develop the *GUS* reporter vectors, the UBQ1 and UBQ2 promoters were amplified from the pLB vector and cloned into the pBI121 vector instead of the 35S promoter via the *Bam*HI and *Hin*dIII sites through homologous recombination (HR) using the ClonExpress II One Step Cloning Kit (Vazyme). To inspect the efficacy of the VvU3 and VvU6 promoters in driving the expression of sgRNA, the grape *PDS* sgRNA was ligated to the VvU3 and VvU6 promoters by PCR, and the VvU3/U6-PDS expression cassettes were cloned into the pCACRISPR/Cas9 vector as previously described^27^. To construct the pCA-UBQ2-Cas9-VvU3/U6-PDS vectors, the *Cas9* gene was amplified from the pCACRISPR/Cas9 vector and cloned into the *Hin*dIII-digested pCAMBIA2300 vector by HR. The UBQ2 promoter was then amplified and cloned into the pCAMBIA2300-Cas9 vector just upstream of the *Cas9* gene via the *Bam*HI and *Sal*I sites through HR. The VvU3-PDS and VvU6-PDS expression cassettes were subsequently amplified from the well-constructed pCACRISPR/Cas9 vectors described above and ligated into the *Sma*I-digested pCAMBIA2300-UBQ2-Cas9 vector via HR. Similarly, to construct the CRISPR vectors for tobacco genome editing, the *NbPDS* sgRNA was fused to the VvU3 and VvU6 promoters by PCR, and the VvU3/U6-NbPDS expression cassettes were introduced into the pCAMBIA2300-UBQ2-Cas9 vector by HR.

To construct the multi-sgRNA/Cas9 vector for multiplex genome editing, the *TMT1* sgRNA and *TMT2* sgRNA were fused to the VvU6.1 and VvU6.2 promoters by PCR, respectively, and the two sgRNA expression cassettes were combined by overlapping PCR. The combined sgRNA expression cassette was cloned into the *Sma*I-digested pCAMBIA2300-UBQ2-Cas9 vector via HR. For construction of the PTG/Cas9 vector, a DNA fragment containing a 50-bp polyA sequence and the polycistronic tRNA-TMT sgRNA expression cassette (Supplementary Fig. S6) was synthesized (Tsingke) and ligated into the *Hin*dIII-digested pCAMBIA2300-UBQ2-Cas9 vector by HR. All the primers used for cloning and vector construction are provided in Supplementary Table S1.

### **P**lant transformation, regeneration, and identification

For the stable transformation of 41B cells, the CRISPR vectors were introduced into the *Agrobacterium* strain EHA105, and *Agrobacterium*-mediated transformation of 41B cells and the regeneration of transgenic plants were conducted as previously described^31^. After transformation, 41B cells were cultured in liquid GM supplemented with 200 mg/L Timentin and 5 mg/L kanamycin, and the grape cells were subcultured every 4 weeks until the development of kanamycin-resistant cells. The resistant cells were sampled for gDNA isolation, and *Cas9*-specific primers (Supplementary Table S1) were used to identify T-DNA insertions. For the regeneration of transgenic plants, 41B cells were transferred to GM without phytohormones. The induced embryos were further germinated on woody plant medium (Duchefa Biochemie) under light conditions^31^. The regenerated plants were sampled for gDNA extraction, and the prepared gDNA was used for the PCR amplification of target fragments. The plants were analyzed by direct sequencing of PCR products followed by sequencing assays of individual amplicon clones^23^. For each plant, a total of 25 clones were analyzed to verify the mutation types.

For transient expression experiments, the CRISPR vectors and *GUS* reporter vectors were introduced into *Agrobacterium* strain GV3101. The bacterial cells were cultured in liquid lysogeny broth medium containing 50 mg/L rifampicin and 50 mg/L kanamycin at 28 °C overnight. The bacterial cultures were centrifuged at 5000 × *g* for 10 min, and the resultant supernatant was discarded, and the bacterial cells were resuspended in transfection buffer (10 mM MES, 10 mM MgCl_2_, and 200 μM acetosyringone, pH 5.6). The concentration of the bacterial suspension was adjusted to a final OD_600_ of 0.4. The suspension was incubated at room temperature for another 2 h before transformation. The leaves of 5-week-old tobacco plants were infiltrated using a 1-mL needleless syringe. Three leaves from three independent plants were harvested as three biological replicates for qRT-PCR assay or GUS staining. For transient expression in grapevine leaves, the leaves of 1-month-old *V. amurensis* plants were immersed in the bacterial suspension, and vacuum was applied for 5–10 min until the leaves became translucent. After infiltration, the grapevine leaves were washed with sterile water and placed on sterile water-soaked filter paper in 90-mm Petri dishes. At least three independent *V. amurensis* plants were used for transformation with each vector. GUS staining of leaves was performed 3 days after infiltration.

### **M**utation detection

For the detection of mutagenesis in the target genes, DNA fragments containing the target sites were amplified by PCR with gene-specific primers from grape or tobacco. gDNA was first treated with the *Ssp*I enzyme to digest the wild-type sequence, and the target sequences of the grape *PDS* gene were then amplified. The PCR products were purified and cloned into the pLB vector for Sanger sequencing. For evaluation of the editing efficiencies, the RFLP method and T7EI assay were performed as previously described^40^. The *PDS* gene contained an *Ssp*I site within the target sequence, and digestion of the PCR amplicons with the restriction enzyme generated undigested bands. The indel frequency was measured based on the intensity of the undigested bands^40^. However, the target sequences of *TMT1* and *TMT2* lacked proper restriction enzyme sites; thus, the T7EI assay was adopted for assessment of the editing efficiencies. The editing-induced mismatched amplicons could be cleaved by the T7EI enzyme, and the indel frequency can be calculated from the intensities of the digested bands^40^. The PCR products treated with *Ssp*I (NEB) or T7EI (NEB) enzyme were separated on an EtBr-stained agarose gel (1.5%). The mutation efficiencies were determined using ImageJ.

### **q**RT-PCR assay

Total RNA was extracted using the HiPure HP Plant RNA Mini Kit, and cDNA was synthesized from 1 μg of total RNA using the HiScript II Q RT SuperMix for qPCR Kit (Vazyme) following the manufacturer’s protocols. qRT-PCR assays were performed to determine the expression levels of the sgRNA and *Cas9* genes in grape cells, and grape *Actin 1* (accession no. AY680701) and *GAPDH* (accession no. XM_002263109) were used as internal controls. In tobacco leaves, the expression of sgRNA relative to that of the *Cas9* gene was measured. The relative expression levels were calculated using the 2^−ΔΔCT^ method^55^. The significance of gene expression was determined using Student’s *t*-test.

### **M**easurement of the sugar content

The content of soluble sugars was determined using an HPLC system as described by Zhang et al.^56^.

### **G**US staining

GUS staining was performed as described by Baltes et al.^57^.

## Supplementary information

Optimizing the CRISPR-Cas9 system for genome editing in grape by using grape promoters
