# Supplementary material for: Optimizing the CRISPR/Cas9 system for genome editing in grape by using grape promoters
Source: Hortic Res. 2021 Mar 1;8:52. doi: 10.1038/s41438-021-00489-z (PMC7917103; doi:10.1038/s41438-021-00489-z)
Supplement: Supplementary file 1 — Optimizing the CRISPR-Cas9 system for genome editing in grape by using grape promoters [file 41438_2021_489_MOESM1_ESM.pdf]

Supplementary Figures and Tables

>VvU3.1
AGTACTTTCATAGGAATAGGTTTCAAATGAACCTTTGTGATACACTTCGATCCTGACTCTCTC
TAAAAGCAAAAATCATTAATATTTTTTCAATTATATTTTAATTTTTACAAATACTAACAAATATT
AATAATTCTAATATCTTTCTTGTTTTGAAAAATAAAAGAAAAATAATAATGTTTGGTATATTCC
GTATATATTATTTTAAATGAATCCAGAAGTTTCCAAGAATTTCACTGGCAATCAATCGTGCAT
CAGCTGTCAATCGTTGTTCCCAGGAAGGCTCATTGGAAGTCTATAACCAATGAGAACACGC
GGTGACTAGCCGTCCCACATCGAAAATGCAGGAAACATTTAATAACTATATAACAAAGGATA
GGAGATTCACATGCC
>VvU3.2
TATTTTTAGTTTTGTTAAAAATAATTATTTTTTAGAAAAGTATTTATAATAAAAATATTATTAAA
AAATATTTTACGAATTAAAAGTACTTTCATAGGAATAGAAAAAAATCATTTACCTTAATTTTT
AAAAAACTTTTACTATGAGATAAACATGATTTTCTCAAAAAAATTTAATCGCAGAATCTTAA
AAATAGGATTCAAGTGACAGATCCGTTACTTTACACGTACAACTATTATCTTCTTTAACGATA
TAAATAAAAAAATGTTTATCAAAATAATTTTGCAAATTAAAATATCATACATCGGCCGTTAAA
ATTATTTTAAATGAATCCAGAAGTTTGCAAGAATTTCATTTGGCAATCTGTCGTGCATCAGCT
GTCAATCGTTGTTACCAAGAAGTTCATTGCAAATATATGGCCAATGAGAACACGCGGTGACT
AGCCGTCCCACATCGGAAATGCAGGAAACATTTAATGACTATATAACAAAGGATAGGAGATT
CACATGCC
>VvU6.1
TTGCCTCTGGAAAATCCCCCTATAATTTTTTCAGTTTCTTTCAATTACCAGCAAAATTAGGGC
TGAGAATGGAATTCCCAAACCCTAAAACAAAAAGAATTCCCAAATCTGAAAACAGTCTAGG
ATTCGGTTTTATAAATTACAAAAAGAACAACCATAATAAAGATGAAAAGCAACGAAGGAAG
AAAAAGGAAATGAGAGAGGAGAGAGGAGACGCAGAGAGAGCAGCAGTCTCACCTTCTGG
TGGGGAACACCAAGGACGAACATGCCAATTCTAAATTCAACCCAAATGAGTTGTGGTGACG
GGCCGTGGGCTCGATGCCCAGACCAAGCGAAACGACGTCGTTCCCAAACGACTCTTCCCA
CATCGACTGCTCATAGACGAAATTGAGCTTTTATATATCAGGAGCAAACGCTTAGAGCTT
>VvU6.2
TTCCCTAATCATCATGTCTCTGCAATCTCTATCTAAGAGCTCCGCAAGAGATGATATTCAAAA
TCTTTTTGCCATGGTTTTGCTTCAAGTTGGTTTTTGATCAGCAGTCAATGGATTTTAAGCTAC
CACCCTCGGTATCCTACATAAGAAATCCAATACAAAAGTGGATTTTTGCAGTGCTGGTAGTT
TCTTGAATTTAAGTTATTTAATTAGACTTATGATAAACACTAGCCCCTGGAAAATTCACCTAC
AATTTTTTTCAGTTTCTTTCAATTACCAGCAAAATTAGGGCTGAGAATGCAATTCCCAAACC
CTAAGAACAAAAAGAATGCCCAAATCTGAAAGCATTATATGACAACAACCATAATAAAGAT
GAAAAGCAACGAAGAAAAAGTAATCTCACCTTCTGGTGGGGAACACCAAGGACGAACATG
CCAATTCTAAATTCAACCCAAATGAGTTGTGGTGACGGGCCGTGGGCTCGATGCCCAGACC
AAGCGAAACGACGTCGTTCCCAAACGACTCTTCCCACATCGACTGCGTATAGACTAAATTC
ACCTTTTTTATATCAGGAGCAAACGCTTAGAGCTT

Fig. S1. The promoter sequences of VvU3.1, VvU3.2, VvU6.1 and VvU6.2.

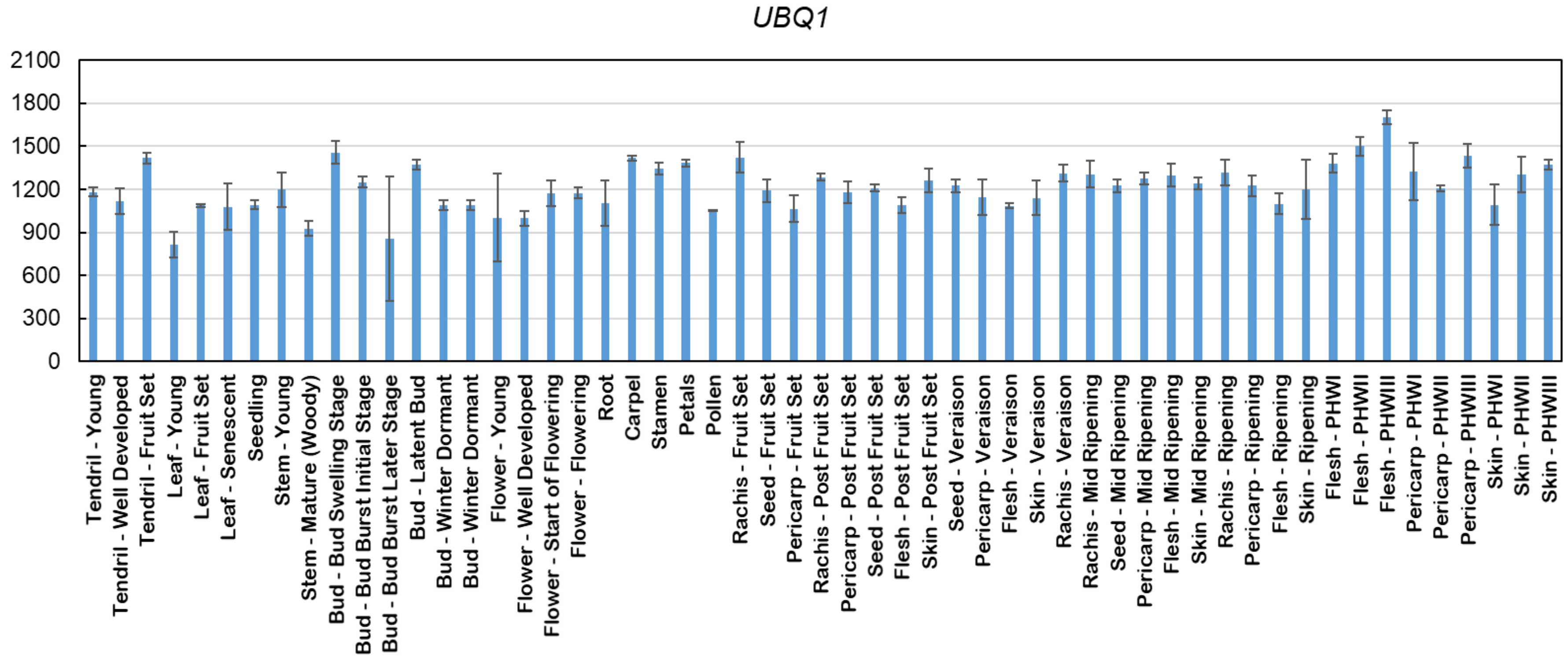
UBQ1
2100
1800
1500
1200
900
600
300
0
Tendril - Young
Tendril - Well Developed
Tendril - Fruit Set
Leaf - Young
Leaf - Fruit Set
Leaf - Senescent
Seedling
Stem - Young
Stem - Mature (Woody)
Bud - Bud Swelling Stage
Bud - Bud Burst Initial Stage
Bud - Bud Burst Later Stage
Bud - Latent Bud
Bud - Winter Dormant
Bud - Winter Dormant
Flower - Young
Flower - Well Developed
Flower - Start of Flowering
Flower - Flowering
Root
Carpel
Stamen
Petals
Pollen
Rachis - Fruit Set
Seed - Fruit Set
Pericarp - Fruit Set
Rachis - Post Fruit Set
Pericarp - Post Fruit Set
Seed - Post Fruit Set
Flesh - Post Fruit Set
Skin - Post Fruit Set
Seed - Veraison
Pericarp - Veraison
Flesh - Veraison
Skin - Veraison
Rachis - Veraison
Rachis - Mid Ripening
Seed - Mid Ripening
Pericarp - Mid Ripening
Flesh - Mid Ripening
Skin - Mid Ripening
Rachis - Ripening
Pericarp - Ripening
Flesh - Ripening
Skin - Ripening
Flesh - PHWI
Flesh - PHWII
Flesh - PHWIII
Pericarp - PHWI
Pericarp - PHWII
Pericarp - PHWIII
Skin - PHWI
Skin - PHWII
Skin - PHWIII

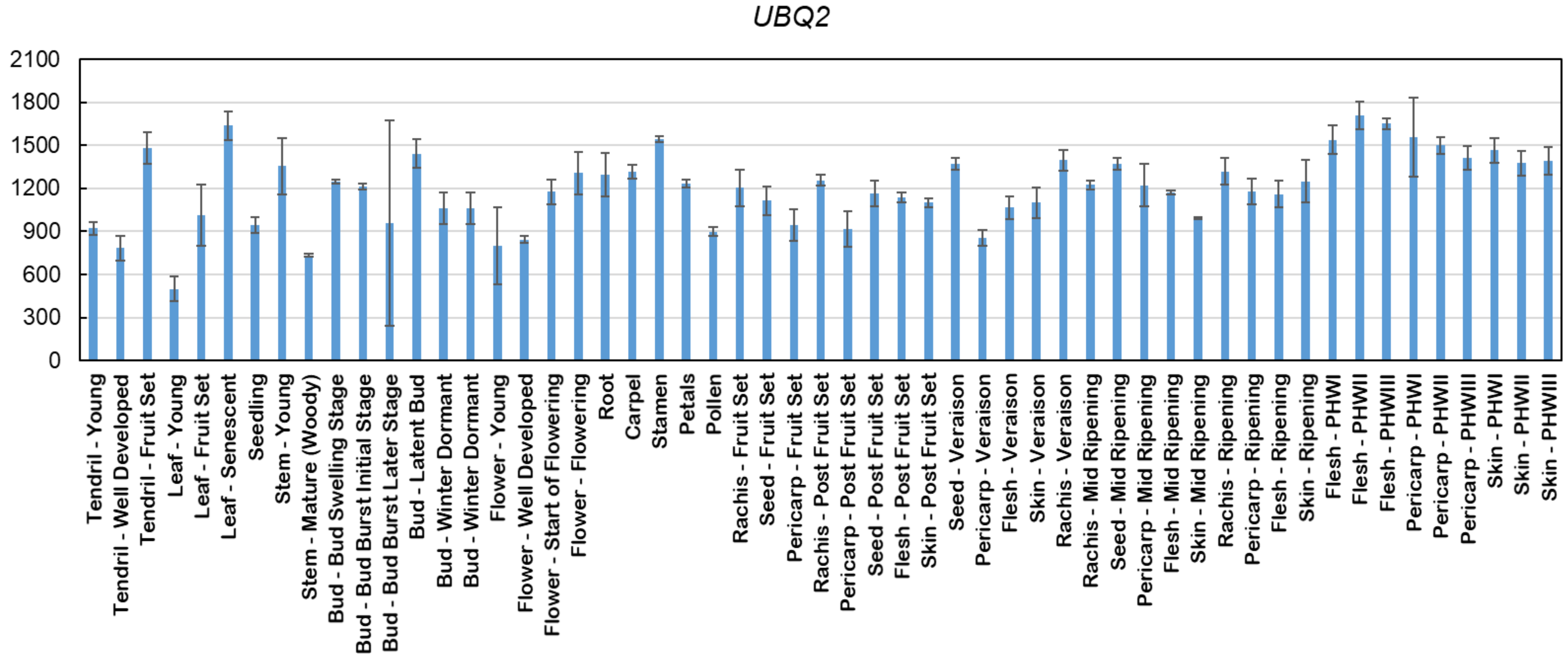

Fig. S2. The expression profiles of *UBQ1* and *UBQ2* genes in grape at different developmental stages. The data were downloaded at the website of Grape eFP Browser (http://bar.utoronto.ca/efp_grape/cgi-bin/efpWeb.cgi).

>UBQ1 promoter
GAAACGGTGACGGAGTCGTTAGCCAGAGACAACTCCGTTACGCCGAGCGTCACA
CCTAACGTCCACCCACCCCCATTTACCAAAGTAATCTCCACTAAATATCCTACCCCA
CCGCCCCCACTTTAACGAACGGCCCAAAATCACCCCCCCCCAAATCCCACCCACA
GAACACCACCGTGATCGTACTCGCCATGAAAACGTGTGGGACTTCTCCTCACCGC
CTAGCACCCTCCGTCATTAAAGTAGAGCCACGAGACATCTAGATGCCGTGGAGGAT
AGGATAAAGGACACGTGGCATCACTCTATTCGTTTATCCTAATCGGTTTCGTCACCG
ACTCGGCAACTGCGACTACAAATAGACGCCCTCTCTTCACAGCCTCTCCATTGTTG
ATACCCTTTTGAAACTACCAGAAAACTCTCATTGCCATCCTTTGAAGCCTTAGAATC
TTTCTTTCAAGGTACCATTCGGTTCTTCTTTGTTTCGTTTTTTATGTTTTGTGTTTTCT
GTTTTTTACAATTCATTGAGTACTTCAGAGAAAATTTCTCCTTCCTTTTCCGAAAGA
ATTTGGATTTCTTTTGATGTTTGTTTGTTTTCTTGTTTTCTTTAAGAGCTTCTTTTTAT
GTGAAGATTTATGGTGCTATTATTGATTGGTTTTGCGAATTCGTAAGGAAAATTTTC
TCCGGTGTAAATCGGTTTTGAAGGTTTTCCTATTTGTTTTTCCCCAGATCGGCTATT
GACGCCCTAAGA

>UBQ2 promoter
GCTGACGGAGTCGTTAGTCATCAACCACTCCGTCACGTGGAGCGTCACAGTTAAC
GTCCGCCAACCCCCATTTACCAAAAAAAACTCCACTAAAAATCCTACCCCACCGCC
CCCACTTATAACGAACGGCCCAAAATCACACTCCACAAATCTCACCCCACAGAAA
CGCACCGTGAACGTACTCGCCACGAACACGTGTGGGACTTCTCCCCCCATCTCCG
TGCTTCAAGTAGAGCCAAGGGACATCTAGATGCCGTGAAGGATAGTGCCAAGAGC
CATCTAGATGCCGTGCAGGAAAGGTTAGCGATACACGTGGCAGAAATCTATTTGCT
ATTTCCTCATCGGTTTCGTCACCGAGTAGGCAACCTCGACTACAAATAGACGCTCT
CACGGGGACTTCTACATCATTGATTTCATATATTCAAGCCATTAGAAAAATCCCTTT
ACCCTTTTGAAGGTACCGTTTGGTTCTTCTTCTATGTTTCGTTTTCTTTTTTGATGCT
TTCTGGTTCTGATTTTTACAATTCATTGAGTAGTCGAGAAAATTTCTCCTTTCTTTGC
CGAAAGAATTTAGATTTCTTTTGATGTTGTTTATATTCTTAATTTTTTGTTAAGGGTT
GCTTTTTATGTGAAAATTTCTGGTATTATTGTTGATTGGTTTTTGCGAAATCGTGAAG
CGAATTTTCTCCGTTGTAGATCGGCTTTTGAAGGTTCTCTTATTCGTTTTTCCTAGAT
CTGGCTATTGATGACTTGAGAATTTGTAGGGAATTTGGGATTTTGGACCTTTTTCAA
TTCTTTTTTCAGATCTGTCGTAATAAATTGGTACTCTCTTAGATCTGTGTAGA

Fig. S3. The sequences of UBQ promoters identified from grape genome.

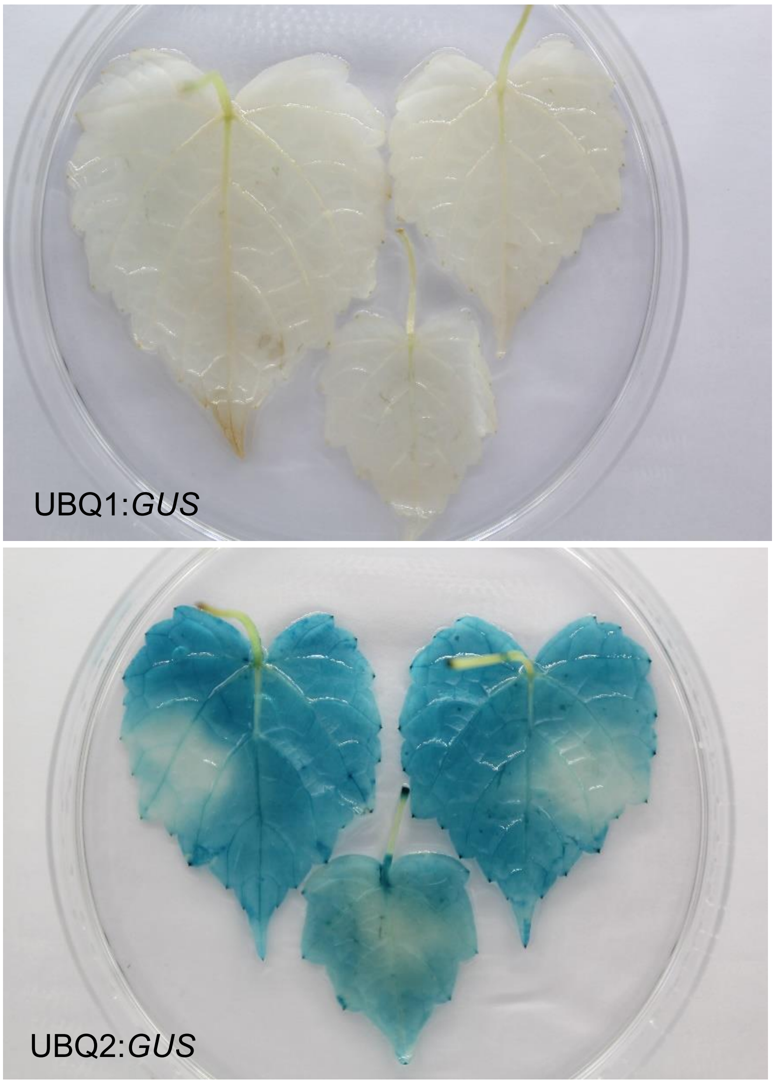

Fig. S4. GUS staining results of *V. amurensis* leaves. The leaves were used for staining three days after infiltration.

```
Ho1    TGGCTGGTTTGTCTACTGCAAAATAATTTGGCAGATGCAGGTCACAAGCC
Ho2    TGGCTGGTTTGTCTACTGCAAA...ATTTGGCAGATGCAGGTCACAAGCC
Ho3    TGGCTGGTTTGTCTACTGCAAAAT...TTGGCAGATGCAGGTCACAAGCC

Bial1 ┌TGGCTGGTTTGTCTACTGCAAAAT...TTGGCAGATGCAGGTCACAAGCC
      └TGGCTGGTTTGTCTACTGCAA..........CAGATGCAGGTCACAAGCC

Bial2 ┌TGGCTGGTTTGTCTACTGC........TTGGCAGATGCAGGTCACAAGCC
      └TGGCTGGTTTGTCTACTGC......ATTTGGCAGATGCAGGTCACAAGCC

Bial3 ┌TGGCTGGTTTGTCTACTGCAAAATAATTTGGCAGATGCAGGTCACAAGCC
      └TGGCTGGTTTGTCTACTGCAAAA..ATTTGGCAGATGCAGGTCACAAGCC

He1   ┌TGGCTGGTTTGTCTACTGC......ATTTGGCAGATGCAGGTCACAAGCC
      └TGGCTGGTTTGTCTACTGCAAAAT.ATTTGGCAGATGCAGGTCACAAGCC

He2   ┌TGGCTGGTTTGTCTACTGCAAAA..ATTTGGCAGATGCAGGTCACAAGCC
      └TGGCTGGTTTGTCTACTGCAAAAT.ATTTGGCAGATGCAGGTCACAAGCC

He3   ┌TGGCTGGTTTGTCTACTGCAAAATAATTTGGCAGATGCAGGTCACAAGCC
      └TGGCTGGTTTGTCTACTGCAAAAT.ATTTGGCAGATGCAGGTCACAAGCC
```

Fig. S5. Genotyping results of grapevine *pds* mutants. The results obtained with some homologous (Ho), biallelic (Bial), and heterozygous (He) plants were provided as examples. The genomic DNA prepared from regenerated plants was used to amplify the target site of *PDS* gene, and the fragments were cloned into pLB vector for sequencing assay. A number of 25 amplicon clones were analyzed for each plant by Sanger sequencing.

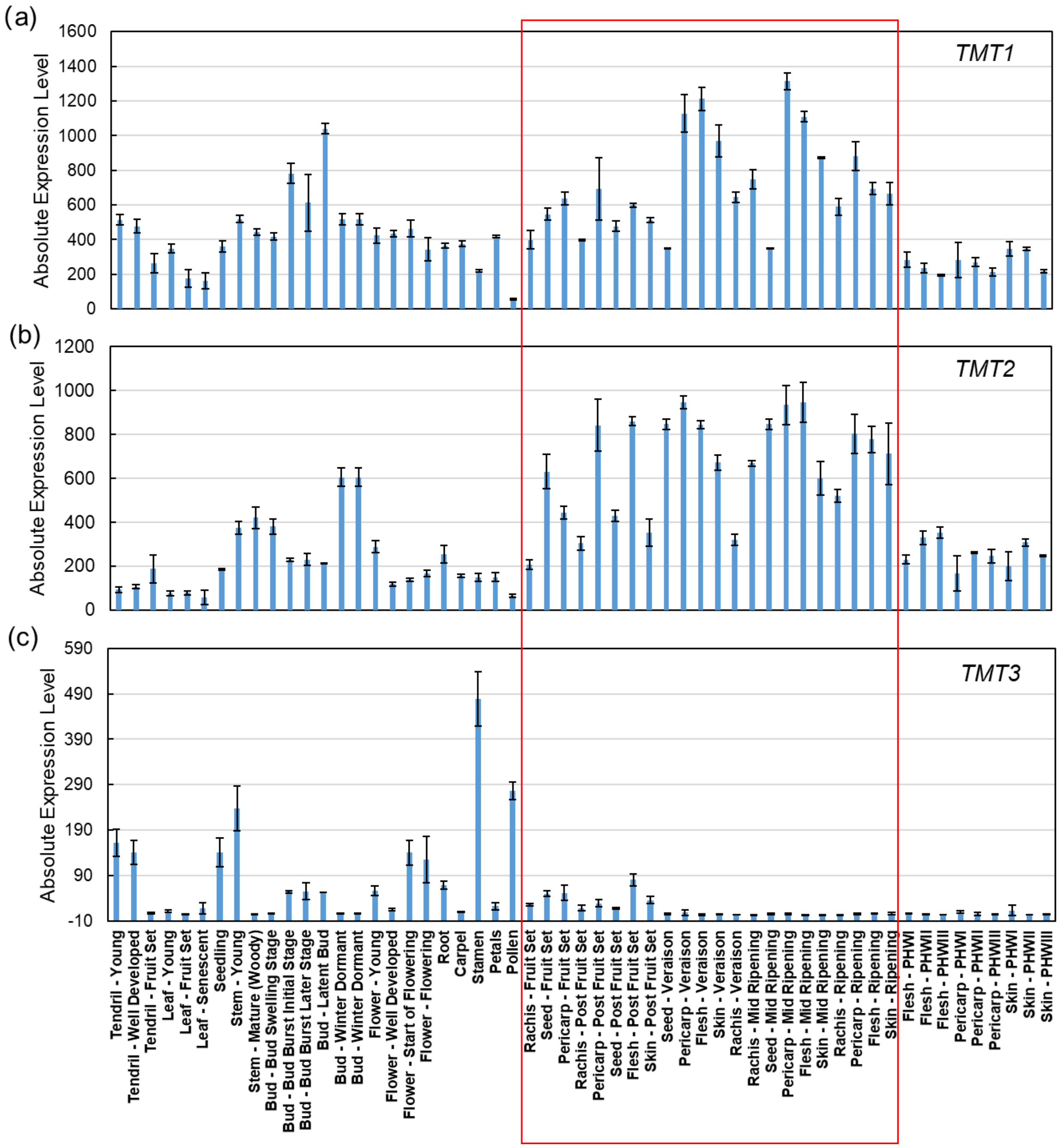

Fig. S6. The expression profiles of *TMT* genes in grape at different developmental stages. The data were downloaded at the website of Grape eFP Browser (http://bar.utoronto.ca/efp_grape/cgi-bin/efpWeb.cgi).

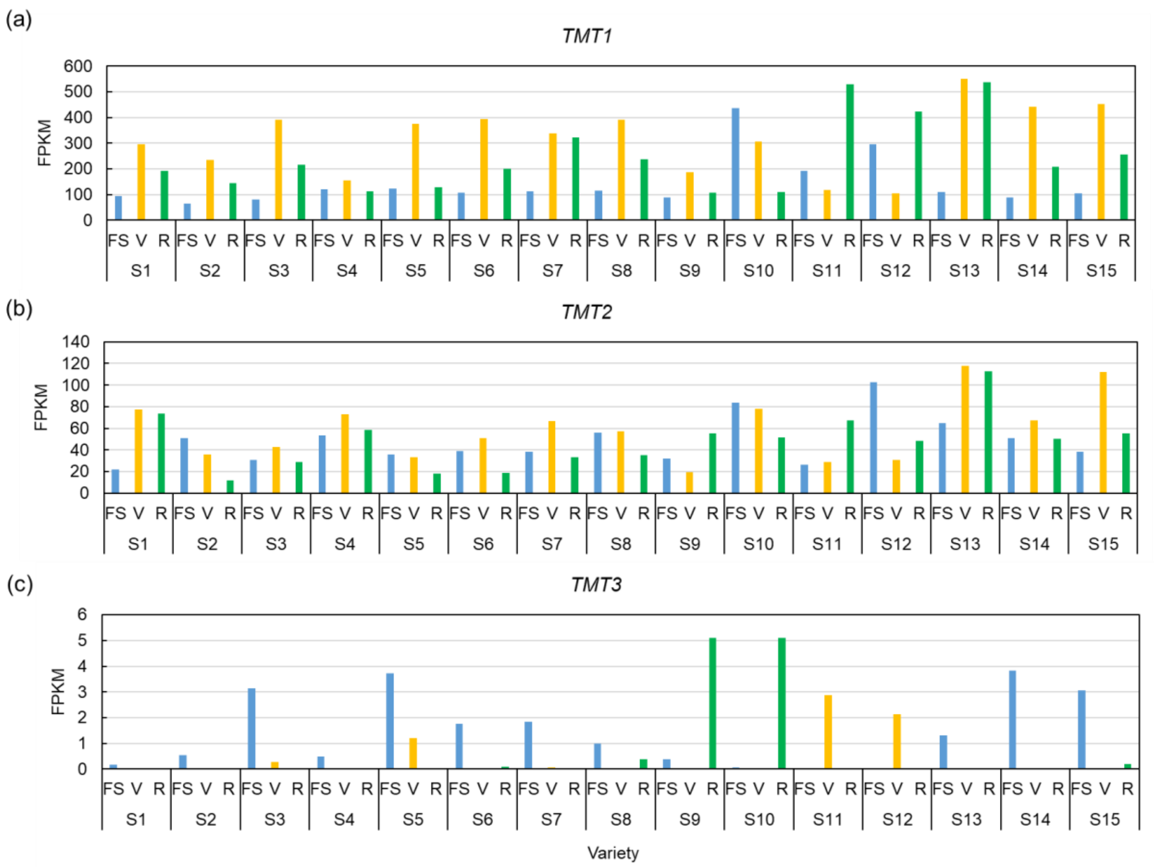

Fig. S7. The expression profiles of *TMT* genes in berries of different grape varieties. The berries at fruit set (FS), veraison (V), and ripening (R) were collected from different varieties (S1-S15) and used for transcriptome analysis (unpublished data). The FPKM values of *TMT* genes were used to evaluate the expression profiles.

.

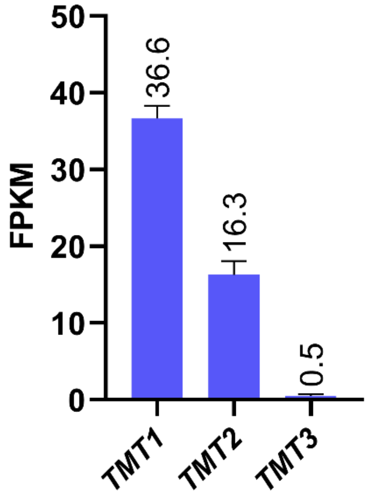

Fig. S8. The expression profiles of *TMT* genes in 41B cells. The wild-type 41B cells were sampled for transcriptome analysis (unpublished data). The FPKM values of *TMT* genes were used to evaluate the expression profiles.

>PTG sequence (PolyA-tRNA-sgRNA)

AAAAAAAAAAAAAAAAAAAAAAAAAAAAAAAAAAAAAAAAAAAAAAA
AAAACAAAgcaccagtggtctagtggtagaatagtaccctgccacggtacagacccgggttcgattcccggctggt
gcaGTGGCTATTGCAGCCGCGATGTTTTAGAGCTAGAAATAGCAAGTTAAAA
TAAGGCTAGTCCGTTATCAACTTGAAAAAGTGGCACCGAGTCGGTGCacaaag
caccagtggtctagtggtagaatagtaccctgccacggtacagacccgggttcgattcccggctggtgcaGCAAGG
TGAACCCACTGTAGGTTTTAGAGCTAGAAATAGCAAGTTAAAATAAGGCTA
GTCCGTTATCAACTTGAAAAAGTGGCACCGAGTCGGTGCacaaagcaccagtggtct
agtggtagaatagtaccctgccacggtacagacccgggttcgattcccggctggtgca

Fig. S9. The synthesized PTG sequence. The polyA, tRNA and target sgRNAs for *TMT1* and *TMT2* are indicated in green, blue and red, respectively.

Table S1. The list of primers used in this study.

Primer name	Sequence (5'-3')	Experiment
VvU3.1-PCR-F	AGTACTTTCATAGGAATAGGTTTCA	Promoter amplification
VvU3.1-PCR-R	CTACAGCACTAACCAACCGA	
VvU3.2-PCR-F	TGATATGAATTGCCCATTTTGTTGA	
VvU3.2-PCR-R	ACTCAACCGTTAATCACGCTCT	
VvU6.1-PCR-F	TTGCCTCTGGAAAATCCCCCTA	
VvU6.1-PCR-R	GACCATTTCTCGATTTGTGCGTG	
VvU6.2-PCR-F	CACTAGGGATGACTGCCTGGA	
VvU6.2-PCR-R	CGCAGGGGCCATGCTAATCT	
VvU3.1-PDS-F	CAGGAAACAGCTATGACCATAGTACTTTCATAGGAATAG	Construction of CRISPR vectors for promoter activity test in tobacco leaves
VvU3.1-PDS-R	GCTATTTCTAGCTCTAAAACAATATTTTGCAGTAGACAAATGGCATGTGAATCTCCTATC	
VvU3.2-PDS-F	CAGGAAACAGCTATGACCATTGATATGAATTGCCC	
VvU3.2-PDS-R	GCTATTTCTAGCTCTAAAACAATATTTTGCAGTAGACAAATGGCCGTAGAGTCAAAG	
VvU6.1-PDS-F	CAGGAAACAGCTATGACCATTTGCCTCTGGAAAATCC	
VvU6.1-PDS-R	GCTATTTCTAGCTCTAAAACAATATTTTGCAGTAGACAAACAAGCTCTAAGCGTTTGC	
VvU6.2-PDS-F	CAGGAAACAGCTATGACCATTTCCCTAATCATCATG	
VvU6.2-PDS-R	GCTATTTCTAGCTCTAAAACAATATTTTGCAGTAGACAAACAAGCTCTAAGCGTTTGC	
VvAtU6-PDS-F	CAGGAAACAGCTATGACCATATTCGGAGTTTTTGTATCTT	
VvAtU6-PDS-R	GCTATTTCTAGCTCTAAAACAATATTTTGCAGTAGACAAACAATCACTACTTCGACTCT	
VvU3.1-NbPDS-F	Same as VvU3.1-PDS-F	Construction of CRISPR vectors for tobacco genome editing
VvU3.1-NbPDS-R	GCTATTTCTAGCTCTAAAACTGGACTCTCAAATTAACGGCTGGCATGTGAATCTCCTATC	
VvU3.2-NbPDS-F	Same as VvU3.2-PDS-F	
VvU3.2-NbPDS-R	GCTATTTCTAGCTCTAAAACTGGACTCTCAAATTAACGGCTGGCCGTAGAGTCAAAG	
VvU6.1-NbPDS-F	Same as VvU6.1-PDS-F	
VvU6.1-NbPDS-R	GCTATTTCTAGCTCTAAAACTGGACTCTCAAATTAACGGCAAGCTCTAAGCGTTTGC	
VvU6.2-NbPDS-F	Same as VvU6.2-PDS-F	
VvU6.2-NbPDS-R	GCTATTTCTAGCTCTAAAACTGGACTCTCAAATTAACGGCAAGCTCTAAGCGTTTGC	
VvU3/U6-PDS-2300-F	TGACTAACGACTCCGTCAGCCAGGAAACAGCTATGAC	Construction of optimized CRISPR vectors for *PDS* editing in grape
VvU3/U6-PDS-2300-R	CGAATTCGAGCTCGGTACCCGGTTCACTAAACCAGCTCT	
SpCas9-2300-F	GACCTGCAGGCATGCATGGACTATAAGGACCACG	
SpCas9-2300-R	GGCCAGTGCCAAGCTGATCTAGTAACATAGATGAC	

UBQ2-2300-F	ATTCGAGCTCGGTACCCGGGGCTGACGGAGTCGTTAGTCA	
UBQ2-2300-R	TAGTCCATGCATGCCTGCAGTCTACACAGATCTAAGAGAG	
VvU6.1-TMT1-F1	TGACTAACGACTCCGTCAGCCCGTTTTACCTGTGGAATCG	Construction of multi-sgRNAs expression cassettes for multiplex editing
VvU6.1-TMT1-R1	ATCGCGGCTGCAATAGCCACAAGCTCTAAGCGTTTGC	
VvU6.1-TMT1-F2	GTGGCTATTGCAGCCGCGATGTTTTAGAGCTAGAAAT	
VvU6.1-TMT1-R2	CGTGGTATGCTAGTTATTGCTCAGCCTCGACCGGAGGAAAATTCCATCCAC	
VvU6.2-TMT2-F1	GTCGAGGCTGAGCAATAACTAGCATACCACGCACTAGGGATGACTGCCTGGA	
VvU6.2-TMT2-R1	CTACAGTGGGTTCACCTTGCCAAGCTCTAAGCGTT	
VvU6.2-TMT2-F2	GCAAGGTGAACCCACTGTAGGTTTTAGAGCTAGAAAT	
VvU6.2-TMT2-R2	CGAATTCGAGCTCGGTACCCGGAGGAAAATTCCATCCAC	
PDS-F	TATGACGCGGCCGAATATCAT	Amplification of target sequences
PDS-R	CCACCTTCACCTGTACTCTTTTG	
NbPDS-F	TTCTACAACTTTAGCATAGTCCACA	
NbPDS-R	CCAGCATCACACTTTCGCAT	
TMT1-F	TGTGGGTGTGCTGGTAGTGA	
TMT1-R	CTCCTGAGCATGTTGTGACCA	
TMT2-F	GGGTGTCTGGATTGAGTGGA	
TMT2-R	ACCCCAAGCATTAACCTCCAG	
Cas9-PCR-F	ACCTTCCGCATCCCCTACTA	PCR identification of T-DNA insertions
Cas9-PCR-R	ATTTCCACGGAGTCGAAGCA	
PDS-gRNA-F	TTGTTTTAGAGCTAGAAATAGCAAGT	qRT-PCR
PDS-gRNA-R	CGACTCGGTGCCACTTTTTC	
NbPDS-gRNA-F	GCCGTTAATTTGAGAGTCCAGT	
NbPDS-gRNA-R	CGACTCGGTGCCACTTTTTC	
Actin1-qPCR-F	CAGCAGATGTGGATCTCAAA	
Actin1-qPCR-R	CTGTGGACAATGGAAGGAC	
GAPDH-qPCR-F	TTCTCGTTGAGGGCTATTCCA	
GAPDH-qPCR-R	CCACAGACTTCATCGGTGACA	
Cas9-qPCR-F	CAGATTCGCCTGGATGACCA	
Cas9-qPCR-R	ATCCGCTCGATGAAGCTCTG	

Table S2. The data of band intensities quantified by ImageJ.

The data of band intensities shown in Fig. 4b

	AtU6			VvU3.1			VvU3.2			VvU6.1			VvU6.2		
	Rep.1	Rep.2	Rep.3	Rep.1	Rep.2	Rep.3	Rep.1	Rep.2	Rep.3	Rep.1	Rep.2	Rep.3	Rep.1	Rep.2	Rep.3
Undigested band	34.633	40.951	49.224	45.617	44.239	36.139	49.163	49.339	44.289	58.655	57.242	56.560	56.097	57.850	63.239
Digested band 1	114.572	123.581	138.441	139.905	134.004	120.938	114.209	115.061	104.838	99.077	97.585	98.290	98.233	103.383	109.566
Digested band 2	131.637	137.491	137.805	114.031	115.333	107.673	128.070	126.376	121.275	104.163	106.371	102.455	103.490	110.011	115.133
Indel (%)	12.33	13.56	15.12	15.23	15.07	13.65	16.87	16.97	16.38	22.40	21.92	21.98	21.76	21.33	21.96

The data of band intensities shown in Fig. 5c

	Multi-sgRNAs/Cas9						PTG/Cas9					
	tmt1			*tmt2*			*tmt1*			*tmt2*		
	Rep.1	Rep.2	Rep.3	Rep.1	Rep.2	Rep.3	Rep.1	Rep.2	Rep.3	Rep.1	Rep.2	Rep.3
Undigested band	27751.522	28690.836	24397.350	24555.380	23928.309	25883.016	34589.258	36198.622	37818.380	30107.309	34106.844	29039.652
Digested band 1	3124.768	3430.816	2998.768	6147.983	7486.104	8857.175	2729.527	3090.163	3408.920	8551.225	11387.125	10264.933
Digested band 2	3383.056	3844.422	3308.812	5919.497	7198.790	7957.983	4185.770	5551.012	6502.305	4056.062	5294.598	5229.184
Indel (%)	10.00	10.68	10.86	19.12	21.28	22.14	10.01	10.15	10.99	16.04	18.05	19.25
